# Hyperfocus: the forgotten frontier of attention

**DOI:** 10.1007/s00426-019-01245-8

**Published:** 2019-09-20

**Authors:** Brandon K. Ashinoff, Ahmad Abu-Akel

**Affiliations:** 1 Department of Psychiatry, Columbia University, 1051 Riverside Dr, New York, NY 10032 USA; 2 Institute of Psychology, University of Lausanne, Quartier UNIL-Mouline, Geopolis, Lausanne, 1015 Switzerland; 3 Centre for Human Brain Health (CHBH), School of Psychology, University of Birmingham, Edgbaston, Birmingham, B15 2TT UK

## Abstract

‘Hyperfocus’ is a phenomenon that reflects one’s complete absorption in a task, to a point where a person appears to completely ignore or ‘tune out’ everything else. Hyperfocus is most often mentioned in the context of autism, schizophrenia, and attention deficit hyperactivity disorder, but research into its effect on cognitive and neural functioning is limited. We propose that hyperfocus is a critically important aspect of cognition, particularly with regard to clinical populations, and that it warrants significant investigation. Hyperfocus, though ostensibly self-explanatory, is poorly defined within the literature. In many cases, hyperfocus goes undefined, relying on the assumption that the reader inherently knows what it entails. Thus, there is no single consensus to what constitutes hyperfocus. Moreover, some studies do not refer to hyperfocus by name, but describe processes that may be related. In this paper, we review how hyperfocus (as well as possibly related phenomena) has been defined and measured, the challenges associated with hyperfocus research, and assess how hyperfocus affects both neurotypical and clinical populations. Using this foundation, we provide constructive criticism about previously used methods and analyses. We also propose an operational definition of hyperfocus for researchers to use moving forward.

## What is hyperfocus?

Hyperfocus, broadly and anecdotally speaking, is a phenomenon that reflects one’s complete absorption in a task, to a point where a person appears to completely ignore or ‘tune out’ everything else. It is generally reported to occur when a person is engaged in an activity that is particularly fun or interesting. An example of hyperfocus is when a child becomes engrossed in a video game to a point where they do not hear a parent calling their name. Although most neurotypical people would likely report experiencing a hyperfocus-like state at some point in their life, it is most often mentioned in the context of autism, schizophrenia, and attention deficit hyperactivity disorder—conditions that have consequences on attentional abilities. Despite the experience of hyperfocus being ubiquitous, in both neurotypical and psychiatric populations, there is very limited explicit academic research into its effect on cognitive and neural functioning. A Google Scholar search (excluding citations) for hyperfocus and variations of the term, namely “hyper-focus”, “hyper focus”, “hyperfocusing”, “hyper-focusing”, and “hyper focusing” in the *title*, returned 6 results. A PubMed search for the same terms in the *title or abstract* returned 19 results. Of these, 7 are empirical studies explicitly focused on assessing cognitive and neural states associated with hyperfocus (in ADHD: Sklar, 2013; Ozel-Kizil et al., 2013, 2016; in schizophrenia: Luck et al., 2014; Sawaki et al., 2017; Kreither et al., 2017; Hahn et al., 2016; Gray et al., 2014). An additional result is a paper that has developed a new questionnaire to assess hyperfocus experiences (Hupfeld, Abagis, & Shah, 2019). We also found 1 study that did not appear in our searches, but was cited in a couple of the papers cited above (Ozel-Kizil, 2013). This naturally leads to a simple question: why is there limited explicit research on an ostensibly common human cognitive and perceptual experience?

In this review, we will attempt to explain why hyperfocus research has been so limited and we will assess if other phenomena in the literature may essentially be hyperfocus going by another name. Based on this, we will propose a clear and testable operational definition of hyperfocus. Finally, we will assess if the definitions of hyperfocus used in the psychiatric literature match our proposed operational definition.

## Why has hyperfocus been forgotten?

There are three major issues to consider. First, there is no clear general or operational definition of hyperfocus in the literature, which usually assumes that the reader inherently knows what it is. As there is limited explicit literature on the topic, references to and descriptions of hyperfocus are typically anecdotal and can differ from paper to paper. Table 1 reflects a comprehensive collection of how the few studies that have explicitly studied hyperfocus have defined it, as well as a representative sample of references to hyperfocus in the ADHD, autism, and schizophrenia literature where it was not the explicit focus of research. For example, Kahl and Wahl (2006) reported that adults with ADHD could “hyperfocus” on activities in which they have special interest but did not define what cognitive or subjective experiences are associated with hyperfocusing. It is important to note that in most of these papers, these are the only references or descriptions of hyperfocus at any point throughout (with some exceptions; See Sklar, 2013), and they rarely provide an operational definition that can be tested. When it is defined, it is rarely operationally defined in a way that can be used for quantitative research. Ozel-Kizil et al. (2013; also see Ozel-Kizil et al., 2014) defined hyperfocusing as being “characterized by intensive concentration on interesting and non-routine activities accompanied by temporarily diminished perception of the environment”. However, this definition raises several questions, such as what defines something as interesting? Does it have to produce enjoyment, like a video game, or is an important homework assignment enough? And how is the perception of the environment diminished? The exceptions to this are the papers in the schizophrenia section of Table 1 (all of which come from the same lab group and use the same operational definition of hyperfocus), but we will discuss these in further detail later in the paper and assess if they provide an appropriate operational definition.

**Table 1:** Various descriptions of hyperfocus from the ADHD, autism and schizophrenia literature

Clinical population	Author(s)	Description of hyperfocus
ADHD	Hupfeld et al. (2019)*	“While the estimated 8 million adults in the USA affected by attention-deficit/hyperactivity disorder (ADHD) might find it nearly impossible to sit still in a lecture hall or excruciatingly challenging to focus on writing a term paper, these same individuals might find themselves spending hours at a time composing a new song, tinkering with their car, writing computer code, or watching television (Kessler et al., 2006). The term “**hyperfocus**” (HF) has been used to characterize this state of heightened, focused attention that individuals with ADHD frequently report (Brown, 2005; Conner, 1994; Ozel-Kizil et al., 2016).”
	Ozel-Kizil et al. (2016)*	“ ‘**Hyperfocusing**’ is defined as a clinical phenomenon of “locking on” to a task in patients with ADHD who have a difficulty of shifting their attention from one subject to another, especially if the subject is about their interests (Conner, 1994). **Hyperfocusing** was mentioned as a state resembling a “hypnotic spell”, according to the subjective experiences of the cases with ADHD (Brown, 2005)… Moreover, **hyperfocused** individuals neglect things other than the condition they are already focused on. Patients with ADHD are reported to be stuck in the activities that they are interested and they keep on doing these things for hours while they lose interest in their surroundings…The patients with ADHD usually report that they cannot understand how the time passes. During **hyperfocusing**, the individuals state that they are aware of the things that they ignore, however they cannot give up what they are doing (Brown, 2005; Conner, 1994). Hyperfocusing is thought to occur on the basis of attention disorder; patients with ADHD have difficulties of focusing and sustaining, as well as shifting their attention.”
	Ozel-Kizil et al. (2013)*	“**Hyperfocusing**, which is characterized by intensive concentration on interesting and non-routine activities accompanied by temporarily diminished perception of the environment, is a clinically well-known phenomenon in patients with attention deficit and hyperactivity disorder. It is also described as ‘locking on’ to some task.”
	Sklar (2013)*	“…**hyperfocus** appears to refer to a more specific (and perhaps extreme) type of sustained attention in which the individual’s behaviour is controlled for a long period of time by a task which is ‘non-routine’ or of interest to him/her, to the point that his/her awareness of the environment is considerably diminished.”
	Goodwin and Oberacker (2011)	“Many children with ADHD have the ability to **hyperfocus** on certain tasks. This trait can confuse parents, as they see their highly distractible child engrossed in a video game, for example. They call his name but he has tuned them out, along with every other stimulus in the room.”
	Schecklmann et al. (2008)	“**Hyperfocusing** is not mentioned in DSM-IV [with respect to ADHD], but it is known from clinical work and can be described as intensive concentration on interesting and non-routine activities accompanied by temporarily diminished perception of the environment.”
	Carver (2009)	“Both research and clinical experience tells us that ADHD Children [sic] can exhibit a type of “**hyperfocus**”—intense concentration and single-minded focus when the activity is very interesting.”
	Kahl and Wahl (2006)	“The researchers noted that “interest” probably the most frequently experience positive emotion, “is an extremely important motivation in the development of skills, competencies and intelligence”. The motivating power of such “interest” may be most apparent when it is absent, as described in the chronic complaints of many adults with ADDs who report that although they can **“hyperfocus”** on activities in which they have special interest, they chronically find themselves unable to mobilize effort for tasks in which they do not feel any special immediate interest, even when they are fully aware that their failure to do that uninteresting task may cause significant problems later.”
Autism	Isomura, Ogawa, Shibasaki, & Masataka, (2015)	“Typically, children with autism are known to… pay abnormal and obsessive attention to detail, and to note and record their environment with exquisite clarity (Casey et al., 2008). They are capable of becoming **hyper-focused** and locked-in on apparently arbitrary subjects of interest, and of sustaining their attention on these subjects for unusually long periods of time…as a result of this internal **hyper-focus**, it would be more difficult for another person to command the attention of the child with autism, and it would also be more difficult for the child himself/herself to command his/her own attention voluntarily (Posner and Dehaene, 1994).”
	Fein (2015)	“The co-existence of strength and vulnerability encapsulated in these narratives captured essential features of the experience of living with Asperger’s Syndrome—a condition that itself brought valued strengths (the ability to **hyperfocus** on a topic of interest, strengths in systematic thinking, an occasionally exquisite sensitivity to sensory input) as well as disabilities.”
	Mayes (2014)	“Unlike most children with ADHD who have difficulty sustaining their focus on anything, children with autism can **hyperfocus** on activities of interest to them (e.g., spending hours twirling a string, assembling puzzles, drawing the same picture over and over, or reading a book).”
	Meilleur, Jelenic, and Mottron, (2014)	“Alternatively, improvements in adaptive abilities may accompany loss of skills involving **hyperfocus**, as autistic people learn and adapt.” (No description of hyperfocus is provided and there is no further mention of it in the paper)
	Bombaci (2012)	“Besides associational thinking and mindblindness, autistic subjects also tend to display extreme concentration when gazing on or thinking about objects that interest them. Other terms for autistic **hyper-focus** are ‘stimming’ and ‘perseveration’. Interestingly, over-selective attention, the clinical term for this form of perceptual difference, echoes William James’s notion of selective attention—a perceptual ability that he associated with masculine power.”
	Mayes, Calhoun, Murray, Ahuja, and Smith, (2011)	“Attention deficit, hyperactivity, and impulsivity are common in children with autism, but, unlike children with ADHD, children with autism have the ability to **hyperfocus** on activities of interest to them, such as spending hours twirling a string or reading a book (Mayes & Calhoun, 1999). Repetitive behaviors in autism (e.g., spinning wheels on a car or drawing the same pictures over and over) are often driven by pleasure…”
	Geurts et al. (2009)	“Difficulties in shifting attention, disengaging attention from details (i.e., **hyperfocus**)”
Schizophrenia	Luck et al. (2019)*	“This new hypothesis states that schizophrenia involves an aberrant ***hyperfocusing*** of processing resources on a small number of representations. In other words, even when the task requires perceiving or remembering multiple objects or locations, PSZ [patients with schizophrenia] tend to focus intensely but narrowly.”
	Sawaki et al. (2017)*	“…the **hyperfocusing** hypothesis, which proposes that PSZ [patients with schizophrenia] tend to focus their processing resources more intensely but more narrowly than HCS [healthy controls] as a result of disrupted attractor dynamics that tend to create deeper basins of attraction and produce exaggerated winner-take-all processing (Luck et al., 2014)”
	Luck et al. (2014)*	“… processing resources are focused more intensely but more narrowly in PSZ [patients with schizophrenia] than in healthy control subjects (HCS [healthy controls]). In other words, PSZ [patients with schizophrenia] focus unusually strongly on some sources of information to the exclusion of others. We call this the **hyperfocusing** hypothesis.”
	Gray et al. (2014)*	“We speculate that impairments in the Divided Attention subtest, and in part also reduced WM [working memory] capacity, reflect an underlying abnormality in the dynamics of local cortical circuits in PSZ [patients with schizophrenia]. Briefly, we propose that an imbalance between excitatory and inhibitory function tends to cause exaggerated local inhibition and an increase in winner-take-all processing. This winner-take-all processing mode is suggested to cause a ‘**hyperfocusing**’ of resources onto a small number of locations or objects, whether they are currently visible (as in the Divided Attention subtest) or being held in memory (as in our WM [working memory] task). When applied to external representations, the tendency to **hyperfocus** may lead to deficits in dividing attention among multiple targets or spreading attention among multiple locations or a broad area in space. When **hyperfocusing** is applied to internal representations, this would lead to a reduction in the number of items, rules, or response alternatives that can be simultaneously active, which could compromise more complex cognitive operations.”
	Leonard et al. (2012)*	“Recent work has instead found that schizophrenia is associated with a ‘failure’ to attend broadly (Elahipanah et al. 2011; Hahn et al. 2012), suggesting that impaired WM [working memory] capacity estimates in PSZ [patients with schizophrenia] may reflect a tendency to **hyperfocus** on a subset of the relevant information rather than an inability to filter irrelevant information.”
	Hahn et al. (2016)*	“…it has been suggested that PSZ [patients with schizophrenia] have a narrowed “attentional spotlight” and difficulty maintaining a wide visual span We followed up on these findings with a visuospatial Allocation Task (SARAT), in which a central cue predicts the location of a peripheral target stimulus… One, 2, or all 4 possible target locations could be cued simultaneously, manipulating the degree to which attention had to be focused narrowly or distributed broadly. Both HCS [healthy controls] and PSZ [patients with schizophrenia] displayed step-wise faster RT with more precise cueing. However, this effect was substantially larger in PSZ [patients with schizophrenia] than in HCS [healthy controls]. Potential explanations for this finding are that (1) PSZ [patients with schizophrenia] “**hyperfocused**” the location to which a predictive cue directed their attention, resulting in disproportionate RT benefits in predictive cue trials, or (2) PSZ [patients with schizophrenia] had difficulty distributing attention broadly, resulting in greater RT costs when there was no advance information about the target location.”
	Prentky (2001)	“Positive symptoms, as Gruzelier and Raine (1994) reported, are associated with higher left than right hemispheric activity, supporting the hypothesis that general overactivation of the left hemisphere or underactivation of the right hemisphere characterizes the C-type. Thus, there is a hypothetical optimal hemispheric imbalance that promotes a constructive, task-specific **hyperfocus** on detail and facilitates problem solving but does not seriously incapacitate or debilitate the individual.”

Entries with an asterisk are papers which explicitly studied hyperfocus

Despite these issues, there appear to be four general features or criteria of hyperfocus that are consistently reported (Table 1):

1. Hyperfocus is characterized by an intense state of concentration/focus.
2. When engaged in hyperfocus, unrelated external stimuli do not appear to be consciously perceived; sometimes reported as a diminished perception of the environment.
3. To engage in hyperfocus, the task has to be fun or interesting.
4. During a hyperfocus state, task performance improves.

Second, it is very difficult to experimentally manipulate a subject into a hyperfocus state (Sklar, 2013). The nature of hyperfocus is such that a person must be completely absorbed in a task that is interesting or fun. However, most cognitive psychology experiments do not meet this requirement. Even if subjects are able to enter into a hyperfocus state with some interesting activity, having them respond to a non-task relevant stimulus will break them out of the hyperfocus state. This prevents monitoring of cognitive functioning while the hyperfocus state is occurring, or at least makes it very difficult to do.

Third, some studies do not refer to hyperfocus by name, but describe processes that appear to be related, such as “in the zone” and “flow”. For example, several researchers (e.g., Esterman, Noonan, Rosenberg, & DeGutis 2012, Esterman, Rosenberg & Noonan 2014; Fortenbaugh et al., 2015; Kucyi, Hove, Esterman, Hutchison, & Valera, 2017) measured performance during a sustained attention task while participants were “in the zone”, a state defined by reduced variability in task performance. Dietrich (2004), on the other hand, assessed the neural correlates of “flow”, defined subjectively as a state of intense concentration with the loss of reflective self-consciousness. However, it is unclear if “in the zone” states, “flow” states, and “hyperfocus” states reflect the same or distinct processes.

This review was motivated by the lack of clarity and consistency across the academic literature with respect to hyperfocus. As such, we intend this paper to address the following issues. First, has hyperfocus been explicitly studied, just under a different name? Therefore, in the next section of this paper, we examine the literature on related phenomena, namely “in the zone” and “flow” states—which at face value appear to instantiate the same subjective experiences and behavioral effects—to evaluate if they reflect a similar or distinct process from hyperfocus. It is important to note here that the notion of “in the zone” reported in this review is distinct from the notion of “in the zone” used in the flow literature—often to describe the flow experience. Here, when we refer to “in the zone”, we are referring to it as defined by Esterman et al. (2012, 2014)—performance characterized by relatively low variability in response times. Notably, this highlights the importance of clarity, specificity, and operational definitions in research. Second, we will propose an operational definition of hyperfocus. And third, as hyperfocus is most often referenced in relation to psychiatric disorders, we will then assess if patients with ADHD, autism, or schizophrenia exhibit hyperfocus-related symptoms relative to our proposed operational definition and if these symptoms are consistent across disorders. The ultimate purpose is to provide a common baseline for researchers of the hyperfocus phenomenon.

## Hyperfocus and possibly related phenomena

### Hyperfocus and flow

Flow was one of the first concepts introduced as part of the field of ‘Positive Psychology,’ which focuses on the “science of positive subjective experience, positive individual traits, and positive institutions” (Seligman and Csikszentmihalyi, 2000, p. 5; also see Seligman, Steen, Park, & Peterson, 2005; Rich, 2013; Lopez and Snyder, 2009). According to Nakamura and Csikszentmihalyi (2009, p. 195–196; also see Nakamura and Csikszentmihalyi, 2002; Csikszentmihalyi 1975, 1997, 2000),

The conditions for entering flow include:

- perceived challenges, or opportunities for action, that stretch but do not overmatch existing skills;
- clear proximal goals and immediate feedback about the progress being made.

Under these conditions, experience seamlessly unfolds from moment to moment and one enters a subjective state with the following characteristics:

- intense and focused concentration on the present moment;
- merging of action and awareness;
- loss of reflective self-consciousness (i.e., loss of awareness of oneself as a social actor);
- a sense that one can control one’s actions; that is, a sense that one can in principle deal with the situation because one knows how to respond to whatever happens next;
- distortion of temporal experience (typically a sense that time has passed faster than normal);
- experience of the activity as intrinsically rewarding, such that often the end goal is just an excuse for the process.

The conditions for entering flow and the subjective experiences associated with it appear to map onto the most commonly reported features of hyperfocus (Table 2). Therefore, we propose that flow and hyperfocus are the same phenomenon. Although we are mindful that just because two phenomena are descriptively similar, they are not necessarily mechanistically identical, there is no evidence to suggest that either flow or hyperfocus are distinct. From our reading of the literature, we note that the psychiatric literature is more likely to use the word hyperfocus and positive psychology literature is more likely to use the word flow, despite the phenomenology being almost identical. With this in mind, the flow literature can be used as a framework to understand hyperfocus. Here, we review the literature on flow in conjunction with cognitive psychology literature on attention with an emphasis on assessing if these literatures can provide insight into hyperfocus, specifically with respect to the common features we identified earlier. We will also review the limited research on the neural correlates of flow to assess the possible neural correlates mediating hyperfocus. Furthermore, if the flow literature provides sufficient evidence of the phenomenology associated with hyperfocus, then we can be more confident that these are the same processes. If not, then we would have to re-evaluate this claim.

**Table 2:** Common descriptive features of hyperfocus and flow, based on the most commonly reported features of hyperfocus and Nakamura and Csikszentmihalyi’s (2009) criteria for flow

Hyperfocus criteria	Corresponding flow criteria/experiences
Hyperfocus is characterized by an intense state of concentration/focus	Intense and focused concentration on the present moment
When engaged in hyperfocus, unrelated external stimuli do not appear to be consciously perceived; sometimes reported as a diminished perception of the environment	Merging of action and awareness
	Loss of reflective self-consciousness (i.e., loss of awareness of oneself as a social actor)
	Distortion of temporal experience (typically a sense that time has passed faster than normal)
To engage in hyperfocus, the task has to be fun or interesting	Experience of the activity as intrinsically rewarding, such that often the end goal is just an excuse for the process
	Perceived challenges, or opportunities for action, that stretch but do not overmatch existing skills
During a hyperfocus state, task performance improves	A sense that one can control one’s actions; that is, a sense that one can in principle deal with the situation because one knows how to respond to whatever happens next

#### Criterion 1: to engage a hyperfocus, the task has to be fun or interesting

Linnell and Caparos (2013; Linnell, Caparos, de Fockert, & Davidoff, 2013, Linnell, Bremner, Caparos, Davidoff, & de Fockert, 2018) have suggested attentional/cognitive engagement, rather than task difficulty (such as in perceptual load theory; Murphy, Groeger, & Greene. 2016; Makovski, Hommel, & Humphreys 2014; Lavie, 1995; Lavie and Tsal, 1994), to be the critical factor in determining the scope of attentional function during a task. Engeser and Rheinberg (2008) examined the effect of perceived importance on flow. Flow was measured with the Flow Short Scale (Rheinberg, Engeser, & Vollmeyer 2002), a ten-item questionnaire designed to assess flow experiences. During a low importance task (playing the video game pac-man), flow was highest when there was equal perceived difficulty of the task and perceived skill at the task by the subjects (termed skills-demand compatibility), compared to when the task was easy or hard. For a high importance task (studying for a university level statistics test that required a passing grade to continue on with the degree program), they found that flow was high when the task was easy and when there was a skills-demand compatibility, compared to when the task was hard. Similarly, Schüler (2007) measured subjects’ achievement motive before and after an academic lecture (with a 1-week interval in between testing sessions) and it was found that those who were motivated by their ‘hope of success,’ as compared to ‘fear of failure,’ experienced flow during the lecture. In both of these studies, increased perceived importance of the task arguably increased the subject’s motivation to engage in it and subsequently moderated the conditions under which flow was achieved. In terms of flow, this suggests that the “experience of the activity as intrinsically rewarding”, may in fact be one of the criteria for entering a flow state, rather than an effect of these states. In terms of hyperfocus, it suggests that engaging hyperfocus requires task engagement, which would simply be more common during fun or interesting tasks.

#### Criteria 2 and 3: intense state of concentration; external stimuli do not appear to be consciously perceived/diminished perception of the environment

In terms of cognitive psychology, we could phrase “intense concentration” as the intense engagement of sustained and selective attention mechanisms. Unfortunately, there are limited cognitive studies of attentional processes in the flow literature. As with the hyperfocus literature, this is largely due to the reasons discussed at the beginning of this review. Although mostly speculative, this section highlights attentional processes that may contribute to flow and hyperfocus. As attention is a limited resource, if a significant amount of those resources are focused on a particular task, peripheral and task irrelevant information may be lost (such as in perceptual load: see references above; and inattentional blindness: Simons and Chabris, 1999; Simons, 2000; Stothart, Wright, Simons, & Boot, 2017). In this case, subjects in a flow state will not pay attention to their own actions beyond what is required for the task, resulting in a ‘loss of reflective self-consciousness’, nor will they be paying attention to the time passing, resulting in ‘the distortion of temporal experience.’ In fact, there is ample evidence that attention is required for conscious awareness (Taylor, 2002; Cohen, Cavanagh, Chun, & Nakayama 2012; Dehaene, Changeux, Naccache, Sackur, & Sergent 2006; but see Lamme, 2003 and Koch & Tsuchiya, 2007 for a dissenting viewpoint) and for accurate time perception (Brown, 1985). When referencing hyperfocus, the literature refers to the “diminished perception of the environment” (Sklar, 2013; Schecklmann et al., 2008), which one could argue may be due to similar mechanisms and result in similar experiences as the effects of flow that were just described. That being said, efficient engagement of attentional resources could also produce an apparent diminished perception of the environment. Crucially, these explanations may not be mutually exclusive. For example, it is possible that attention is efficiently engaged such that external distractors could be processed and suppressed, but time perception is still impaired due to a lack of available resources. A corresponding set of effects could also occur with respect to information that is relevant to the task. In fact, the well-known phenomenon of the attentional blink (Raymond, Shapiro, & Arnell, 1992; Dux and Marois, 2009; Shapiro, Hanslmayr, Enns, & Lleras 2017) shows that under conditions of significant attentional engagement even centrally presented, task relevant information may be ignored.

Unfortunately, this way of thinking about flow has rarely been applied in an experimental context. In a notable exception, Castellar, Antons, Marinazzo, and Looy (2019; also see Allison and Polich (2008) had participants engage in an auditory novelty oddball task while simultaneously playing a video game under three conditions (manipulated by adjusting the difficulty of the game): boredom, frustration, and flow. The results showed that the participants made more errors in the oddball task during the flow condition, than boredom or frustration, suggesting they were focused more intently on the video game during the flow state (measured with a questionnaire given after the task) and that they did not perceive “external” stimuli (relative to the video game) as efficiently. This study also included EEG measurements, but that will be discussed further in the next section. Future research will have to continue to investigate the relationship between flow and cognitive functioning with sophisticated cognitive paradigms like this.

#### Criterion 4: task performance improves

It is critical to ask ‘Does flow/hyperfocus actually improve performance?’ Csikszentmihalyi and colleagues (Csikszentmihalyi, 1997; 2000; Nakamura and Csikszentmihalyi, 2009) have consistently described flow as a state that produces inherently high performance, yet there is limited evidence to support this statement. Keller, Bless, Blomann, and Kleinböhl (2011); Keller, Ringelhan, and Blomann (2011) conducted a study in which subjects were presented with a computerized knowledge task; they were presented with a question and had to select an answer from four response options. There were three separate experimental conditions: boredom, fit, and overload. In the boredom condition, the questions were consistently very easy throughout the blocks. In the fit (or adaptive) condition, the difficulty of the questions was adapted based on performance (when they got a question right, the next one was harder; when they got one wrong, the next one was easier). It was predicted that the fit condition would produce a flow state. In the overload condition, the difficulty of the questions was consistently too hard for the subjects throughout the blocks.^1^ After the knowledge task, subjects filled out a questionnaire indicating if they experienced flow or felt that skills-demand compatibility was met. Throughout this study, heart rate variability (HRV) was measured as a dependent variable, with the logic that a low HRV was indicative of mental involvement and/or strain. Subjects reported being ‘more involved’ with the fit condition than the boredom and overload conditions. Additionally, it was reported that HRV was significantly decreased in the fit and overload condition compared to the boredom condition. The difference in HRV between fit and overload conditions was not significant.^2^ A second experiment following a similar procedure, but measuring salivary cortisol (an indicator of stress), found that subjects produced more cortisol in the fit and overload conditions compared to the boredom conditions. There was no difference between the overload and fit conditions. It was reasoned that increased stress would be expected in the overload condition, as subjects would be struggling to succeed, but that the same levels of stress in the fit condition is surprising. Keller et al., (2011) argued that despite the generally positive subjective experience associated with flow, “flow experiences can be considered as involving straining tension and mental load from a physiological perspective”. This can be interpreted that the brain is in fact ‘working harder’ than normal, lending credence to the idea that people might perform better during a flow state. However, these studies only provide indirect and speculative evidence that performance might increase during a flow state because one’s brain appears to be working harder.

Other studies have also provided more, albeit still indirect, evidence of increased performance in flow states (Schüler 2007; Jin, 2012; Engeser and Rheinberg, 2008; Keller and Bless, 2008). Keller and Bless (2008) had subjects play the video game *Tetris* under varying difficulty conditions (Adaptive, Boredom, and Overload) and found that subjects in the adaptive condition (where subjects purportedly were in a flow state) had higher top scores than subjects in the other conditions. This was interpreted as evidence that flow experiences result in improved performance. Additionally, Schüler (2007; experiment 2) reported that flow experience^3^ was a significant predictor of exam performance, such that more intense flow experiences were associated with higher grades. However, as all of this evidence is correlational, causal relationships cannot be inferred. In fact, Jin (2012) has suggested that the direction of the relationship between performance and flow may be the opposite; her study found that high performance while playing a video game was a predictor of experiencing flow.

Based on this, it is unclear if task performance actually improves during flow or hyperfocus states. One possibility is that flow states simply make people feel as if they were doing better than usual, rather than actually improving performance. The flow experiences of the ‘merging of action and awareness’ and having ‘a sense that one can control one’s actions’ may be explained by the fact that more attentional resources are deployed towards the relevant information than is typical, resulting in faster processing of information (Carrasco & McElree, 2001; Carrasco, Giordano, & McElree, 2006; Noguchi, Tanabe, Sadato, Hoshiyama, & Kakigi 2007). According to the ease-of-processing heuristic (Kornell, Rhodes, Castel, & Tauber, 2011), information that is processed quickly is judged to be learned better than more slowly processed information. In addition, Winkleman and Cacioppo (2001; see also Reber, Schwarz, & Winkielman 2004) showed that faster processing of information results in higher positive affect. This could give subjects the impression that they always know what to do because they are making quick judgments that they feel good about, regardless if they are correct. Of course, this will require explicit testing in the future.

#### The neural correlates of flow

Research into the neural correlates of flow is limited. Dietrich (2004) proposed a theoretical framework based on the idea that information processing consists of an explicit (i.e., conscious, voluntary) and implicit (i.e., unconscious, automatic) system. In this framework, flow is associated with suppression of activity in the explicit system, specifically the frontal and prefrontal cortex—referred to as “transient hypofrontality”. According to Dietrich (2004), this allows the implicit network to be engaged without interruption, producing flow states. However, Weber, Tamborini, Westcott-Baker, and Kantor (2009) point out that Dietrich’s (2004) framework does not account for flow as an attentional phenomenon (see below for an alternative framework). To our knowledge, only one study has explicitly investigated the neural correlates of flow in a healthy population. As described in an earlier section. Castellar et al. (2019) had participants engage in an auditory novelty oddball task while simultaneously playing a video game during a flow state and, based on the behavioural data from the oddball task, ostensibly less intense attentional states (boredom and frustration). During the task, EEG measurements were recorded, and they found a delayed (by 24 ms on average) midfrontal negativity during the flow condition, which was interpreted as “a neural correlate of executive attentional processes involved in top-down cognitive control operations… reflecting executive processes as defined in the three-network view of attention… (p. 9)”. Moreover, Raz and Buhle (2006) reviewed fMRI research on the three attention networks (alerting, orienting, and executive) and reported that the alerting and executive function networks were both independently associated with increased frontal cortex activity, notably in the dorsolateral prefrontal cortex and the anterior cingulate cortex. Other non-frontal brain regions have also been associated with attentional processes and executive control, such as the parietal cortex and subcortical regions, but we focus on frontal regions here in response to the transient hypofrontality framework. Therefore, if we posit that flow (or hyperfocus) is first and foremost an attentional phenomenon, then these results may contrast Dietrich’s (2004) notion of “transient hypofrontality” as the trigger for flow states.

That being said, we urge caution in inferring attentional processes based on increased or decreased frontal activation, a rationale known as reverse inference. There is some debate on the value of both formal and informal reverse inference in neuroimaging data (Sprooten et al., 2017; Poldrack 2011; Yarkoni, Poldrack, Nichols, Van Essen, & Wager, 2011; Poldrack, 2006) and a full treatment of this is beyond the scope of this paper. However, most relevant to our purposes is that Poldrack (2011) has argued that reverse inference is useful as a method to generate novel hypotheses. In other words, we cannot say based on the current literature that increased frontal activation in alerting and executive functions contradicts transient hypofrontality during flow states, because frontal activity does not inherently imply the engagement of attentional control, but the literature does provide enough evidence to justify the development of a study designed to test this possibility. And we would encourage readers to do just that!

Another theoretical framework was proposed by Weber et al. (2009), suggesting that flow states are the result of synchronization between attentional (particularly alerting and orienting) and reward networks—states that can be induced when there is a balance between skill and challenge in a task. They argue that synchronization has fewer metabolic demands than non-synchronous brain activity, that it is a particularly efficient method of information processing, that it is a mechanism for conscious awareness, and that it manifests subjectively as a pleasurable experience. It should be noted that Weber et al. (2009) provided this definition specifically in the context of media, such as video games. The available empirical data relating to this theory is both limited and mixed. Only one study we found attempts to explicitly test this theory. Klasen, Weber, Kircher, Mathiak, and Mathiak (2012) showed activation in the reward and alerting network in response to the skill/challenge balance during a video game, arguing that it supported the notion of synchronization between reward and attention areas. Although critically, this is indirect evidence as they did not obtain any explicit measures of functional connectivity or synchronization. However, other studies that were not explicitly designed to test the synchronization theory, nevertheless provide contradictory evidence. For example, Kucyi et al. (2017) found that, during a rhythmic tapping task, increased functional connectivity between and within the default mode network (DMN) and the salience network was associated with “out of the zone” attention. To the extent that “out of the zone” contrasts “the flow state” (see discussion in following section), this interpretation contradicts the notion that increased synchronization is associated with more efficient information processing. Future research will have to resolve these conflicting results.

### Hyperfocus and being “In the Zone”

In an effort to assess moment to moment fluctuations in sustained attention performance, Esterman et al. (2012) developed the gradual continuous performance task (grad CPT). During the grad CPT, participants were presented with images of either a city scene or a mountain scene that would gradually transition from one to the next over the course of the 800 ms presentation time. Participants had to respond to city scenes and withhold responses during mountain scenes. To assess behavioral variation in performance over time, Esterman et al. (2012) employed a variance time course analysis, which assessed within-subject performance. They found that high variability epochs were associated with increased errors, faster RTs prior to commission errors, and slower RTs prior to correct responses relative to low variability epochs. As such, the low variability epochs were termed “in the zone” and the high variability epochs were termed “out of the zone”. One significant advantage of the variance time course analysis was that it allowed for the measurement of attentional states without having to directly probe the subjects to find out if they were “in the zone”.

Esterman et al. (2014) conducted a similar study using the grad CPT paradigm, but also included irrelevant distractors. In this study, participants had to respond to male faces, while withholding responses to female face. These faces were superimposed on top of mountain or city scenes which independently transitioned out-of-sync with the faces. On some trials, the background image was the same as the previous trial (repeat trials) and on others it changed (novel trials). Behaviorally, they reported that “in the zone” states were associated with fewer errors, suggesting improved task performance. Moreover, using a cortical-scene network ROI, regions that are selectively activated by scene images, they found a larger difference in the BOLD % signal change between novel and repeat trials (with more activity during novel trials) during “in the zone” epochs relative to “out of the zone” epochs. Because scenes were the distractors in their study, they argued that this was evidence of increased distractor processing, which was possible because of a concomitant reduction in perceptual load associated with the primary face identification task. Essentially, they argue that “in the zone” epochs are characterized by efficient attentional engagement, while “out of the zone” epochs are characterized by over engagement.

Based on these data, there do seem to be similarities between hyperfocus and “in the zone” epochs. Notably, the evidence suggests that “in the zone” states reflect intense engagement of sustained attention and reduced distraction. Critically, although numerous studies have described hyperfocus as being associated with a temporarily diminished perception of the environment (Table 1; Sklar, 2013; Ozel-Kizil et al., 2013; Schecklmann et al., 2008) resulting in fewer distractions, Esterman et al. (2014) would suggest that it may be associated with an enhanced perception of the environment leading to more efficient distractor suppression. However, despite the apparent similarities, “in the zone” states do not meet the criteria to be classified as the same process as hyperfocus. First, the task is not especially fun or interesting beyond a traditional psychology experiment. And second, the nature of the variance time course analysis is such that there is no baseline condition, so it is unclear if performance during the “in the zone” epochs reflects enhanced performance or simply unimpaired performance, relative to impaired performance during the “out of the zone” epochs.

## An operational definition of hyperfocus

Based on the above review, we propose the following operational definition for four distinct and testable features of hyperfocus: (1) hyperfocus is induced by task engagement; (2) hyperfocus is characterized by an intense state of sustained or selective attention; (3) During a hyperfocus state, there is a diminished perception of non-task relevant stimuli; and (4) During a hyperfocus state, task performance improves.

Flow states do show evidence that they are induced by at least interesting, if not fun tasks. In particular, they are induced by engaging tasks, irrespective of the source of motivation. Although the specific language is different from what has been typically used to describe hyperfocus (engagement vs fun/interesting), it is arguable that in context they mean the same thing. There is no explicit evidence that flow induces intense states of sustained and selective attention. However, based the reported effects of flow, it is reasonable to hypothesize that sustained and selective attention likely play a significant role. Moreover, applying a cognitive framework can provide a reasonable account of how intense states of attention could lead to the reported effects of flow. There is ample evidence that flow states induce a “diminished perception of non-task related stimuli”, though this has been only reported in subjective questionnaires, after the flow state had ended. To date, there have been no explicit cognitive or psychometric measurements taken during a flow state. Regardless, the subjective reports match the anecdotal reports of hyperfocus closely. Finally, there is some limited evidence that flow states improve task performance, but this is all correlational and indirect. Overall, the evidence suggests that flow states and hyperfocus appear to be the same phenomena, just with different names and initially reported in different fields of psychology. That being said, more research will be necessary to confirm and strengthen this claim (Table 3).

**Table 3:**
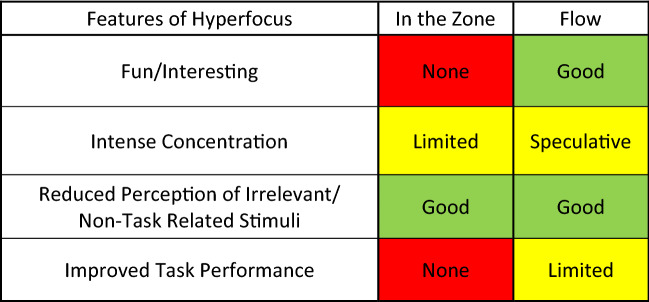
Evidence for the phenomenology of hyperfocus during “in the zone” or “flow” states (color figure online)

The colored boxes indicate the quality and quantity of the evidence relating to different features of hyperfocus. In the online version, green indicates good evidence, yellow indicates limited or speculative evidence, and red indicates no evidence. In the print version, white indicates good evidence, light grey indicates limited or speculative evidence, and dark grey indicates no evidence

“In the zone” states show no evidence that they are induced by fun or interesting tasks. They do show some evidence that they are characterized by intense states of sustained attention, but it is unclear how “intense” these states really are. “In the zone” states seem to reflect the upper range of normal fluctuations in attentional performance, rather than a distinct state of attention. “In the zone” states also show clear evidence of increased distractor suppression, resulting in a diminished perception of non-task relevant stimuli. Finally, there is no evidence that “in the zone” states result in improved task performance. Overall, the evidence leads us to conjecture that while “in the zone” states may still be mediated by similar mechanisms to hyperfocus, they are nevertheless distinct phenomena. More research will be needed to investigate this possibility (Table 3).

## Hyperfocus in psychiatric disorders

References to hyperfocus most frequently arise in research on ADHD, schizophrenia, and autism. Each disorder is reported to increase the frequency and/or magnitude of hyperfocus states, sometimes in different contexts. This phenotypic overlap may not be surprising given evidence for a genetic overlap across the three conditions (Cross-Disorder Group of the Psychiatric Genomics Consortium, 2013). For each population, we will review how hyperfocus has been measured, the general consensus on how hyperfocus affects these populations, and we will provide constructive criticism about the methods and analyses used in each. There will also be a discussion about whether the measures of hyperfocus used across populations reflect the same process or not.

### Hyperfocus and ADHD

Attention deficit hyperactivity disorder (ADHD) is characterized by clinically significant (i.e., it interferes with daily life) hyperactivity, impulsivity, and inattention. It should be noted that, despite its seemingly self-descriptive name, ADHD is not solely a disorder of attention, but also executive functions (Roberts, Martel, & Nigg, 2017; Castellanos, Xavier, Sonuga-Barke, Milham, & Tannock 2006; Willcutt, Doyle, Nigg, Faraone, & Pennington 2005). Moreover, there are three subtypes of ADHD (inattentive, hyperactive, and combined), which may even be distinct disorders (Milich, Balentine, & Lynam 2001; Roberts and Milich, 2013). According to the DSM-V (APA, 2013), one of the symptoms of ADHD is that the child “often does not seem to listen when spoken to directly”. While the DSM-V does not explicitly refer to this symptom (or any other symptom for that matter) as hyperfocus, references to it as a symptom of ADHD are pervasive in academic literature (Goodwin and Oberacker, 2011; Travis, 2010; Carver, 2009; Kahl and Whal, 2006; Ozel-Kizil et al., 2013; Schecklmann et al., 2008; Sklar, 2013; see also Table 1). Indeed, Hupfeld et al. (2019) found that patients with ADHD experience hyperfocus more often than healthy, neurotypical controls both in general and across a range of specific settings (in school, during hobbies, during “screen time”, and in the “real world”). In addition, although hyperfocus is seemingly antithetical to the association of ADHD with inattention and impulsivity, it is often reported as a positive state in individuals with ADHD because they actually engage in tasks for longer periods of time than is typical (Goodwin and Oberacker, 2011; Travis, 2010).

Research into hyperfocus and ADHD is extremely limited. We were only able to find one study that explicitly attempted to measure cognitive and neural differences in hyperfocus between ADHD and neurotypical populations. In this study, Sklar (2013) took EEG measurements while ADHD and neurotypical participants played a first-person shooter game, ostensibly measuring brain activity during a hyperfocus state. In this study, hyperfocus was essentially defined to be identical to flow, as described by Csikszentmihalyi (1997, 2000). There were a few important findings. First, patients with ADHD showed reduced alpha and beta levels in the frontal lobe relative to controls while playing the game; and although not significant (but mentioned in light of small sample sizes) alpha and beta levels decreased over the course of the game for the ADHD patients, but increased for the controls. This was interpreted as evidence that ADHD patients required less cognitive effort to play the game, in line with the reported experiences of hyperfocus. Second, in the frontal midline, delta wave activity increased significantly over the course of the game (and it was reported that theta wave activity increased at a trend level; *p* < .10). It was speculated that this might reflect the “experience of the activity as intrinsically rewarding” element of hyperfocus. Third, in the parietal lobe, the mean absolute power was higher in the ADHD patients than the controls. This was notable because typically ADHD patients show lower parietal activation than controls, which is thought to reflect impaired attentional process in ADHD. Sklar (2013) argued that these results supported the notion that impairments to attention may be context-specific in patients with ADHD. In other words, it is possible that patients with ADHD are not impaired when in a hyperfocus state and may even have enhanced attentional control. And fourth, a post-experiment questionnaire revealed that patients with ADHD experienced a more distorted perception of time, possibly supporting the notion that they did in fact experience hyperfocus during the task.

However, some methodological aspects need to be considered for a better understanding of the results. First, there is a question of whether or not the participants (both ADHD and neurotypical) experienced hyperfocus while playing the games. Sklar (2013), like Weber et al. (2009), argued that media, such as video games, provided the appropriate environment to induce hyperfocus/flow states. Noteworthy, Weber et al.’s (2009) framework is predicated on the notion that hyperfocus is the result of enhanced neural synchronization between attentional and reward networks in the brain. However, Hoekzema et al. (2014)showed that patients with ADHD exhibited reduced functional connectivity between the dorsolateral pre-frontal cortex and various brain networks, and notably the DMN, during attention demanding tasks. Additionally, Querne et al. (2014; see also Fassbender, Scangos, Lesh, & Carter 2014) reported that, in contrast to neurotypical participants, children with ADHD did not show significant anti-phase synchronization (a form of inhibitory synchronization) between the DMN and task positive networks (TPN; brain regions that activate during “externally oriented” task—including the dorsal and ventral fronto-parietal attention networks). This was interpreted as an impairment in the ability of the TPN to suppress the DMN due to immaturity in ADHD-related brain development, even in adult ADHD (Castellanos and Elmaghrabi, 2017; Catellanos et al., 2006; Kelly, Margulies, & Castellanos 2007; Scheres, Milham, Knutson, & Castellanos, Scheres et al., 2007). Based on this evidence, it is questionable whether Weber et al.’s (2009) synchronization-based theory of hyperfocus is likely, and by extension if simply playing games is enough to consistently induce hyperfocus across subjects. That being said, if attention deficits are contextual, as suggested by Sklar (2013), then perhaps the connectivity deficits identified by Hoekzema et al. (2014) and Querne et al. (2014) would not be found if the task induced hyperfocus.

Second, Sklar (2013) did not assess behavioral measures that might correspond with neurological measurements. Although this was an understandable methodological choice, the use of a video game provides a unique opportunity because performance over the course of the game can theoretically be measured (for example, most games have some kind of scoring mechanism) without having to probe the subject (admittedly, this is easier said than done). In general, it is good practice to include behavioral measurements to compare to neurological measurements, so as to be able to establish a relationship between brain activity and behavior (i.e., linking hypotheses; Teller, 1984; Morgan, Melmoth, & Solomon, 2013).

ADHD is synonymous with a high degree of distractibility and having a short attention span. However, the oft reported hyperfocusing states in this condition suggest that individuals with ADHD can, paradoxically, sustain attention excessively. In fact, attentional control may not be as impaired in patients with ADHD as once thought. For example, Roberts, Ashinoff, Castellanos, and Carrasco (2018) have shown that spatial covert attention is functionally intact in adults with ADHD. Therefore, sophisticated investigation of the nature of hyperfocus in ADHD is critically important as it may provide important etiological clues that have been previously overlooked due to a focus on “distractibility”. Moreover, despite the ubiquity of reports of hyperfocus in patients with ADHD, it is not reflected in the DSM criteria for a diagnosis. Perhaps this should be reconsidered since, based on anecdotal evidence, hyperfocus appears to be a core symptom.

### Hyperfocus and autism

Autism spectrum disorders (ASD) are neurodevelopmental disorders associated with impairments in social development, language, and repetitive, circumscribed behaviors/interests. Two defining symptoms of ASD are “(B1) Stereotyped or repetitive motor movements, use of objects, or speech (e.g., simple motor stereotypies, lining up toys or flipping objects, echolalia, idiosyncratic phrases)” and “(B3) Highly restricted, fixated interests that are abnormal in intensity or focus (e.g., strong attachment to or preoccupation with unusual objects, excessively circumscribed or perseverative interest)” (APA, 2013). Of particular interest here are the cases where individuals with autism exhibit an intense focus on a particular behavior or topic, which are sometimes explicitly referred to as hyperfocus (although usually anecdotally; Mayes, 2014; Clark, 2016; Fein, 2015; Bombaci, 2012). The term hyperfocus is also sometimes used to refer to stereotypic behavior or stimming (short for self-stimulatory behavior; Bombaci, 2012—see Table 1). However, these are distinct phenomena that co-occur in ASD and need to be treated as such, with hyperfocus referring to symptom B3 and not B1, although we recognize that the two sets of symptoms may be difficult to tease apart from a phenomenological and clinical perspective. Here, we focus on studies that appear to get at the phenomenon of hyperfocus in ASD, rather than stereotyped behaviors or stimming.

To our knowledge, there are no studies that specifically attempt to measure behavior or cognitive performance during hyperfocus or flow states in ASD. In a review paper, Geurts et al. (2009) defined hyperfocus in the context of ASDs as “difficulties in shifting attention, disengaging from details”. So, it might be surprising to learn that some fundamental attentional processes appear to be intact in patients with ASD, including exogenous and endogenous spatial attention (Grubb et al., 2013a, b) and attentional disengagement (Fischer, Plessow, Dreisbach, & Goschke 2014), although other aspects of attention have been shown to be deficient. For example, Keehn, Westerfield, Müller, & Townsend, (2017) found that children with ASD, unlike typically developing children, showed no behavioral or electrophysiological evidence of attentional capture.

Geurts, Corbett, and Solomon (2009) also proposed that hyperfocus in ASD was associated with cognitive flexibility (the ability to re-allocate cognitive resources based on the situation; Brady et al., 2017; South, Ozonoff, & Mcmahon, 2007; Hill and Bird, 2006; Lopez, Lincoln, Ozonoff, & Lai, 2005). The neural correlates of cognitive flexibility in ASD are usually assessed using attention or task switching paradigms, such as the Wisconsin card sorting test (WCST; Yeung, Han, Sze, & Chan 2016) or reversal learning tasks (D’Cruz, Mosconi, Ragozzino, Cook, & Sweeney, 2016). In these tasks, individuals with ASD have exhibited hypoactivation in the prefrontal cortex, striatum, and parietal cortex (Shafritz, Dichter, Baranek, & Belger, 2008; Gilbert, Bird, Brindley, Frith, & Burgess 2008; Yeung et al., 2016; D’Cruz et al., 2016; Gomot et al., 2006), which is thought to reflect impairments in the ability to shift between different behaviors. Important to our purposes, this hypoactivation has been associated with stereotyped behaviors, which may be confounded with hyperfocus. However, D’Cruz et al. (2016) noted that hypoactivation in a reinforcement learning task was specifically associated with unpredictable task outcomes. They suggested that uncertainty may induce anxiety in patients with ASD, motivating them to maintain behaviors with predictable outcomes and resist novelty. Thus, it is unclear if ASD-related deficits in cognitive flexibility are related to hyperfocus, stereotyped behaviors, or other processes.

Overall, it appears as if hyperfocus is a real phenomenon that occurs in ASD, but care must be taken to distinguish it from other symptoms such as stereotypic behaviors. Research into hyperfocus in ASD is important because, as of now, it is unclear if hyperfocus is a primary symptom or a secondary symptom that is merely induced by other ASD-related behaviors. It is possible that mechanisms underlying hyperfocus behaviors are not autism-specific, but rather that ASD behaviors happen to trigger the same kind of hyperfocus seen in the general population more frequently or strongly.

### Hyperfocus and schizophrenia

Schizophrenia is a disorder characterized by abnormal social behavior, deficits in emotional processing, and psychosis. Symptoms of schizophrenia are divided into three categories: positive symptoms, negative symptoms, and cognitive dysfunction (APA, 2013). Positive symptoms are those that individuals with schizophrenia can experience that are not present in the healthy population. These include hallucinations, delusions, and other forms of psychosis. Negative symptoms are deficits in cognition or emotion in individuals with schizophrenia that are not impaired in the healthy population. These include, but are not limited to, anhedonia, flat affect, and lack of motivation. Cognitive dysfunction refers to deficits across a wide range of cognitive abilities. In the last few years, a hyperfocusing hypothesis of schizophrenia has been developed (see Luck, Hahn, Leonard, & Gold, 2019 for a review of this hypothesis; Luck et al., 2014; Gray et al., 2014; Hahn et al., 2016; Sawaki et al., 2017; Kreither et al., 2017). Here, hyperfocus is defined as the use of processing resources more intensely (i.e., stronger working memory representation), but more narrowly compared to healthy control subjects.

Luck et al. (2014; Gray et al., 2014) showed that a colored distractor that matched a color held in working memory had a greater distracting effect during saccadic eye movements in individuals with schizophrenia than in healthy controls. They argued that the individuals with schizophrenia had generated a more intense working memory representation of the color because they had focused more intensely (i.e., hyperfocused) on it. Additionally, Leonard et al. (2013) showed that the neural mechanisms underlying the working memory differences between individuals with schizophrenia and healthy controls are not the same as those that underlie general individual differences in working memory. They argued this reflected a deficit in the ability of individuals with schizophrenia to distribute their attention broadly. Though the authors are careful to note that attributing these results to hyperfocusing is a conjecture, they do suggest that it provides converging evidence for such a theory.

More recent studies have also supported the hyperfocusing theory of schizophrenia. Sawaki et al. (2017) found that individuals with schizophrenia showed electrophysiological evidence of abnormal attentional focus towards goal-relevant stimulus features. Participants had to respond when a centrally located circle matched a pre-defined target color, while ignoring two colored distractor circles that horizontally flanked the central circle. On some trials, when the central circle was a non-target color, one of the distractor circles could match the target color (i.e., a goal-relevant feature embedded in a distractor). To account for frequently reported deficits in goal maintenance, the target color was presented constantly between trials throughout the experiment. They found that on these trials, neurotypical controls exhibited a significant distractor positivity event related potential (ERP) component, a measure of attentional suppression. This suggested that attention was not directed towards the distractor containing goal-relevant information. However, the individuals with schizophrenia exhibited a significant N2pc (N2 posterior-contralateral) ERP component, a measure of spatial attention shifts and focus towards a lateralized stimulus. This suggested that they actually focused their attention (or hyperfocused) on the distractor containing goal-relevant information. The authors argued that this was evidence that individuals with schizophrenia hyperfocused on goal-relevant information when maintaining a task set. This is consistent with some of the most commonly reported features of hyperfocus, particularly improved task performance. In fact, Beck et al. (2016) reported that, as a consequence of hyperfocusing on goal-related information, people with schizophrenia showed significantly better performance on a probabilistic visual search task.

Kreither et al. (2017) addressed another prediction of the hyperfocus hypothesis, namely that individuals with schizophrenia would focus attention more narrowly, in addition to more intensely. They interpreted this to mean that hyperfocus would be strongest for stimuli in central vision, but weak in peripheral vision. Participants had to discriminate between standard and oddball stimuli at either central or peripheral locations. They found, based on an abnormal P3b ERP component (a measure of higher-level processing resources), individuals with schizophrenia were able to suppress peripheral stimuli when they were responding to centrally located stimuli, but could not suppress central stimuli when they were responding to peripheral stimuli. The healthy controls exhibited the opposite pattern of results. Moreover, they showed that the P3b results correlated with performance in the useful field of view task (UFOV), which measures distributed attention. Individuals with schizophrenia showed worse performance than controls.

Considering the symptom variability associated with schizophrenia, a natural question is whether hyperfocus is associated with negative symptoms, positive symptoms, or cognitive dysfunction. Luck et al. (2014) tested for, but did not find, a correlation between hyperfocusing and the severity of positive (BPRS; Faustman and Overall, 1999) or negative (SANS; Andreasen, 1989) symptoms in their individuals with schizophrenia. However, they obtained a single subscale score for each symptom type (positive and negative) and did not assess if individual symptoms were associated with hyperfocus. If only a subset of positive (like delusions) or negative symptoms are associated with hyperfocus or hypersalience, then a generalized subscale score may not be sensitive enough to reflect these effects. Luck et al. (2019) reported that there is an association across several studies between cognitive dysfunction and the intensity of hyperfocus. Furthermore, they proposed that hyperfocus may be a cause of cognitive dysfunction, rather than a consequence, but are careful to note that this is speculative. They further note that it is unclear, due to a lack of research in unmedicated patients and patients with current psychotic symptoms, if hyperfocus is associated with the positive and/or negative symptoms of schizophrenia. Prentky (2001) argued that patients that exhibit positive symptoms show “higher left than right hemispheric activity. Thus, there is a hypothetical optimal hemispheric imbalance that promotes a constructive, task-specific hyperfocus on detail”. There is also evidence that patients with schizophrenia show a greater preoccupation with delusional beliefs than healthy controls (Sisti et al., 2012), which could be interpreted as hyperfocusing on the belief. In fact, many descriptions of individuals with schizophrenia report the experience of hallucinations being distracting and engrossing, even to the point of exhaustion (Walsh, Hochbrueckner, Corcoran & Spence, 2016; Flanagan et al., 2012). This suggests that individuals with positive symptoms are more likely to experience hyperfocus, or at least that positive symptoms may induce hyperfocus.

More generally, although we do not doubt the results or value of the “hyperfocusing theory of schizophrenia”, it is debateable if its operational definition of hyperfocusing reflects the same process that is typically described in anecdotal reports of hyperfocusing. The hyperfocusing theory does appear to reflect intense concentration and improved task performance. And, although it is generally consistent with reports that people who hyperfocus “tune everything else out”, it is not clear if, in anecdotal reports, people were always focused on stimuli at the center of vision. A future study will need to assess if hyperfocus can only occur at the center of vision or the loci of hyperfocus can move, such as with the spotlight of attention. Moreover, there was nothing particularly fun or interesting about the tasks. A further consideration was that the effect of “hyperfocus” was relatively consistent over time in these studies, which is inconsistent with the notion that hyperfocus reflects an irregular, inconsistent state of attention that is difficult to induce. One possibility is that the “hyperfocus theory” studies were tapping into a visual attention counterpart of hypersalience (rather than hyperfocus) that has been previously reported in individuals with schizophrenia in decision-making tasks. Speechley, Whitman, and Woodward (2010) showed that individuals with delusions exhibited a bias for evidence that matched their expectations, which may explain the results of Luck et al. (2014).

### Is hyperfocus the same phenomenon across psychiatric conditions?

Does hyperfocus, reported in the ADHD, autism, and schizophrenia literature, refer to the same phenomenon in all three conditions? Before we can address this, we must first consider more generally if these disorders present with similar symptoms.

*ADHD and autism* Panagiotidi, Overton, and Stafford (2017; also see Kern, Geier, Sykes, Geier, & Deth, 2015; Banaschewski et al., 2005) investigated co-occurring traits in autism spectrum disorder (ASD) and ADHD. They assessed 334 healthy, neurotypical participants with two ADHD questionnaires and two ASD questionnaires. Their most relevant finding for our purposes was a moderate positive correlation between the attention switching subscale of the autism quotient (AQ; Baron-Cohen, Wheelwright, Skinner, Martin, & Clubley 2001) and the inattention subscales from both ADHD questionnaires (The Wender Utah Rating Scale and the Adult ADHD Self-Report Scale; Ward, Wender, & Reimherr 1993; Kessler et al., 2005). They defined hyperfocus as “difficulties in diverting attention between tasks” and inattention as “difficulty in sustaining attention”, suggesting that these phenomena were reflected in the questionnaire results. They suggested that ADHD and ASD may share a common etiology, and that hyperfocus and inattention may be related to a common mechanism. Therefore, ADHD and autism present with similar symptoms, possibly including a similar form of hyperfocus.

*ADHD and schizophrenia* Research has shown that ADHD and schizophrenia present with similar cognitive deficits in executive functions and attentional function (Banaschewski et al., 2005). In fact, ADHD and schizophrenia cohorts are frequently used as psychiatric control groups for each other. However, there has been no explicit comparison of hyperfocus in these groups. That being said, it is possible that both ADHD and schizophrenia present with a similar form of hyperfocus.

*Autism and schizophrenia* According to Crespi and Badcock (2008), “psychosis and autism represent two extremes on a cognitive spectrum with normality at its center. Social cognition is thus underdeveloped in autism, but hyper-developed to dysfunction in psychosis”. Recent studies have provided support for this spectrum account showing opposite attentional effects based on the relative expression of autism and psychosis in healthy participants (Abu-Akel et al., 2016a, b, c, 2018) and in patient populations (Abu-Akel et al., 2018). In comparing attentional set shifting, Abu-Akel et al. (2018) showed that children with autism had difficulties with extra-dimensional shifts, and children with schizotypal disorders had difficulties with intra-dimensional shifts. Based on this, it seems unlikely that patients with autism and schizophrenia would exhibit a similar form of hyperfocus, since their attentional control patterns seems to reflect a diametric relationship.

However, an alternative interpretation is that patients with these disorders simply hyperfocus on different types of stimuli, making it difficult to see a direct comparison. Crespi and Backcock (2008) conceptualized symptoms of ASD and schizophrenia related to under- and over-mentalizing (i.e., capacity for theory of mind), respectively (see also Abu-Akel and Bailey, 2000; Frith, 2004). In this conception, patients with ASD under-interpret social cues, leading to social withdrawal which would arguably result in focusing on things rather than people, and patients with schizophrenia over-interpret social cues, leading to symptoms like paranoid delusions, which is interpretable as a focus on people and their intentions. Langdon and Brock (2008), however, argued that this interpretation is incomplete as patients with schizophrenia in particular exhibit both over- and under-mentalizing. Furthermore, using a social judgement task, Stanfield et al. (2017) showed that deficits in social cognition in ASD and schizophrenia are mediated by distinct neural correlates. Thus, although additional research is needed to confirm if phenomenon like hyperfocus is expressed via similar mechanisms in these disorders, it does not appear likely.

*Are they the same?* Based on the evidence, we propose that the version of hyperfocus described in this review and more generally in anecdotal reports, occurs in both ADHD and autism, although in autism the term is often incorrectly used to refer to stereotypic and self-stimulatory behavior. The evidence for hyperfocus in schizophrenia is less clear. Although there is a so-called “hyperfocus theory of schizophrenia”, we propose that the effects they have identified reflect hypersalience rather than hyperfocus. Aside from these studies, references to hyperfocus in the schizophrenia literature are few.

## Conclusions

This review discussed the well-known, but poorly investigated phenomenon of hyperfocus, which is referenced in the ADHD, autism, and schizophrenia literature. Until now, there has been no clear operational definition of hyperfocus, leading to a wide range of behaviors being referred to as hyperfocus. To eliminate confusion in the future, we provided a clear and testable definition of hyperfocus that comprised four criteria: (1) to engage in hyperfocus, the task has to be engaging (i.e. fun, interesting, important, etc.). (2) Hyperfocus is characterized by an intense state of sustained or selective attention. (3) When engaged in hyperfocus, there is a diminished perception of non-task relevant stimuli. (4) During a hyperfocus state, task performance improves. Although this definition may change in light of future research, it provides a reasonable starting point for the investigation of this phenomenon. Moreover, we proposed and provided supporting evidence for the notion that the phenomenon of flow is synonymous with hyperfocus. Last, we reviewed the references to hyperfocus in the psychiatric literature to determine if they fit with our newly established definition. We propose that the hyperfocus referenced in the ADHD and autism literature does fit with our definition, but the hyperfocus referred to in the schizophrenia literature does not.

In all, the purpose of this review was to bring attention to an important, but generally forgotten or ignored phenomenon that may be a critical element in several psychiatric disorders. We hope that our operational definition of hyperfocus will facilitate the development of new research paradigms that are specifically tailored to assess the hyperfocus state and underlying mechanisms in both healthy and psychiatric populations. One possibly fruitful avenue for future research is to explore the potential of gamification of cognitive psychology paradigms combined with assessments of performance over the course of the task (like in Esterman et al., 2012, 2014). This may allow researchers to meet the necessary criteria to both induce hyperfocus states, assess when a hyperfocus state is occurring in time, and measure cognitive performance during these time periods.

## Footnotes

1 Some details of the study were not reported by the authors. For example, the threshold criterion in the staircase procedure was not reported nor was if the overload condition adapted to subjects responses.

2 The authors reported that HRV for overload and fit differed at ‘trend level’ significance (p < .10), which is an invalid interpretation that is reported properly here. However, because of this, they interpreted HRV as being uniquely low in the fit condition and do not address the serious theoretical implications of there being no significant difference between the fit and overload conditions.

3 It is unclear from the paper if the ‘flow experience’ that influenced exam performance occurred over the class lectures or during the exam itself.
